# Primary Basal Cell Carcinoma of the Conjunctiva

**DOI:** 10.7759/cureus.31516

**Published:** 2022-11-14

**Authors:** Kah Ling Low, Yin Peng Lai, Rohanah Alias, Jemaima Che Hamzah

**Affiliations:** 1 Department of Ophthalmology, Universiti Kebangsaan Malaysia, Kuala Lumpur, MYS; 2 Department of Ophthalmology, Hospital Kuala Lumpur, Kuala Lumpur, MYS; 3 Department of Ophthalmology, Faculty of Medicine, Universiti Kebangsaan Malaysia, Kuala Lumpur, MYS

**Keywords:** wide excisional biopsy, conjunctival growth, pigmented conjunctival lesion, ocular surface squamous neoplasia, basal cell carcinoma

## Abstract

Epithelial conjunctival malignancies are one of the most prevalent ocular surface tumors. Primary basal cell carcinoma (BCC) of the conjunctiva is extremely rare. We report the case of a 67-year-old Indian gentleman who presented with a fleshy conjunctival lesion for one year on his right eye. Examination revealed a lightly pigmented conjunctival mass adjacent to the limbus. The surface was irregular and non-ulcerated with few feeder vessels. The working diagnosis was ocular surface squamous neoplasia (OSSN). A wide excisional biopsy using the no-touch technique and double-freeze-thaw cryotherapy to the conjunctival margins was performed. The bare scleral area was covered with an amniotic membrane. Histopathological examination revealed a basaloid cell neoplasm favoring BCC, and a tumor-free margin was achieved. Three cycles of topical mitomycin-C 0.02% were prescribed as adjunct chemotherapy postoperatively. There was no evidence of recurrence three months after treatment. The primary BCC of the conjunctiva is unusual and can resemble OSSN. Therefore, it should be considered in the differential diagnosis of patients presenting with atypical features of OSSN.

## Introduction

Conjunctival tumors include a spectrum of benign and malignant neoplasms arising from the conjunctival epithelium, conjunctival stroma, and structures within the stroma, including the blood vessels, nerves, fat, and lymphoid tissue. The most frequent invasive epithelial tumors of the ocular surface are ocular surface squamous neoplasia (OSSN) [1]. On the other hand, primary basal cell carcinoma (BCC) arising from the conjunctiva is incredibly uncommon. In this report, we provide the clinical illustration of a fleshy conjunctival BCC mimicking an OSSN.

## Case presentation

**Figure 1:**
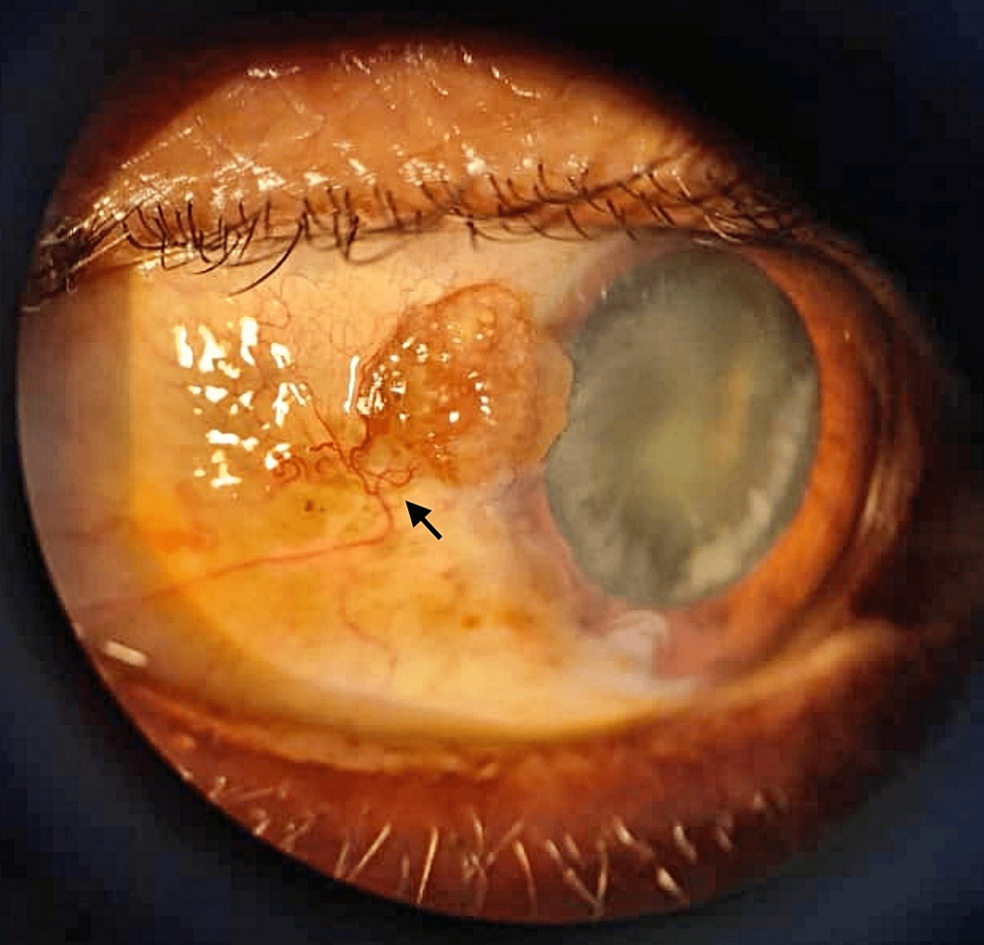
Clinical appearance of the tumor involving the temporal conjunctiva adjacent to the limbus. The lesion is lightly pigmented, raised, and surrounded by feeder vessels (arrow).

A 67-year-old Indian gentleman presented with a fleshy conjunctival lesion for one year in his right eye (Figure 1).

He was a construction worker with long hours of sun exposure. He had no comorbidity, history of trauma, or history of malignancy.

Examination revealed a raised, lightly pigmented conjunctival nodular lesion measuring 3 mm (height) × 3.5 mm (width) × 3 mm (length) located at the 10 o’clock position adjacent to the limbus on the right eye. The surface was irregular, fleshy, and non-ulcerated with prominent feeder vessels. Leukoplakia and papillomatous signs, which are frequently seen in OSSN, were not present. The posterior segment of both eyes was normal. There was no evidence of systemic involvement or involvement in the adjacent structures, including the cornea, caruncle, plica semilunaris, or eyelid. The working diagnosis was OSSN.

A wide excisional biopsy using the no-touch technique and double-freeze-thaw cryotherapy to the conjunctival margins was performed. The bare scleral area was covered with an amniotic membrane. Histopathological examination revealed a basaloid cell neoplasm favoring BCC with positivity for p53, p63, CD10, and BCL2 and negativity for BerEP4 and EMA immunohistochemically. An adequate surgical free margin was achieved, and the patient was prescribed three cycles of topical mitomycin-C (MMC) 0.02% postoperatively. Follow-up examination over three months after completion of MMC showed no evidence of tumor recurrence.

## Discussion

BCC develops almost exclusively on hair-bearing skin, especially on the face due to chronic exposure to sunlight. It accounts for 85-95% of all malignant tumors of the eyelid [2]. BCC has rarely been described to arise as a primary tumor of the conjunctiva. It is far more common to observe eyelid BCC with secondary involvement of the conjunctiva [1]. In the literature, only five reports of primary conjunctival BCC have been illustrated [3-7].

Of the five primary conjunctival BCC reported in the literature, male cases (n = 4) were more common than female cases (n = 1) [3-7]. Patient ages ranged from 60 to 82 years. All cases described the tumor as a single, unilateral, nodular lesion located in the actinically exposed interpalpebral conjunctiva. The most common location was the limbus (n = 3), followed by the medial canthal region (n = 2). The site in the present case (interpalpebral conjunctiva and near the limbus) and the age and sex of our patient are typical of the condition. Table 1 summarizes the features of previously reported cases compared with the present case.

**Table 1:** Summary of previously reported cases of primary basal cell carcinoma of the conjunctiva. M = male; F = female; L = left; R = right; MMC = mitomycin-C

Studies	Sex and age	Eye, L/R	Location on the conjunctiva	Appearance	Treatment	Follow-up data
Aftab and Percival [3], 1973	M, 82	Unspecified	Nasally in the palpebral aperture between the plica and limbus	Pedunculated, fleshy, mobile, 4 mm	Excision	No recurrence for 2 months
Apte et al. [4], 1975	F, 69	R	Nasally in the palpebral aperture between the plica and limbus	Pedunculated, fleshy, irregular margin, and lobulated surface	Excision	No recurrence, but period not specified
Husain et al. [5], 1993	M, 66	L	Nasal limbus	Nodular, fleshy, vascular	Excision	No recurrence for 12 months
Cable et al. [6], 2000	M, 69	L	Temporal limbus	Elevated nodule, 6 mm × 6 mm	Enucleation	No systemic metastasis
Mudhar et al. [7], 2019	M, 60	L	Temporal limbus	Black hemispherical nodule with feeder vessels	Wide local excision with free margin, double-freeze cryotherapy with MMC, followed by topical MMC 0.04%	No recurrence for 2 months
Present case, 2022	M, 67	R	Temporally in the palpebral aperture near the limbus	Elevated, lightly pigmented nodule with prominent surrounding vessels	Wide local excision with free margin, double-freeze cryotherapy, followed by topical MMC 0.02%.	No recurrence for 3 months

Causative factors for the development of BCC in the conjunctiva are unclear. Several mechanisms have been postulated. Metaplasia of the conjunctival epithelium secondary to ultraviolet damage or development of BCC from a dermoid choristoma has been suggested in causing BCC in the conjunctiva [7]. Furthermore, it may be metastasized from adjacent skin cancer [7]. Nevertheless, prolonged sun exposure can be a contributing factor in our patient who revealed no systemic or skin malignancies.

It is challenging to diagnose primary conjunctival BCC clinically due to its rarity. Occasionally, it can mimic other conjunctival tumors and leads to misdiagnosis. Aftab and Percival reported a primary BCC of the conjunctiva resembling a papilloma clinically [3]. In one report, BCC appeared as a dark brown pigmented mass near the limbus and was thought to be a conjunctival melanoma [7]. Our patient had a unilateral vascularized pigmented limbal mass located in the sun-exposed interpalpebral fissure laterally, clinically mimicking an atypical OSSN. Based on our provisional diagnosis, wide excisional biopsy and double-freeze cryotherapy to conjunctival margins were carried out and confirmed the diagnosis of BCC.

The management of OSSN involves surgical removal using the no-touch technique and non-surgical treatments including topical chemotherapy (MMC, 5-fluorouracil), topical/injection immunotherapy (interferon alpha-2b), topical antiviral medication (cidofovir), or photodynamic therapy [8]. Comparatively, treatment for primary conjunctival BCC has not been formally established. The absence of recurrence after tumor excision in previous reports [3-5] may suggest BCC can be adequately managed by complete excision of BCC with a tumor-free surgical margin. In a recent case report by Mudhar et al. [7], intraoperative cryotherapy and postoperative topical MMC 0.04% were administered despite the complete excision of the tumor clinically and histopathologically. Evidence-based research on the role of adjunctive therapeutic modalities in primary BCC of the conjunctiva is still lacking. Nevertheless, our patient who received intraoperative cryotherapy and postoperative topical MMC 0.02% showed no evidence of tumor recurrence over three months after completion of MMC.

Although BCC is a slow-growing tumor, rarely, it can be aggressive locally and can possibly spread intraocularly. In one of the previous reports [6], tumor cells invaded the episclera, sclera, limbus, ciliary body, trabecular meshwork, and anterior chamber after two years of the initial presentation. Enucleation was performed, and the oncology workup for systemic metastasis was negative. As in our case, the lesion was growing slowly for one year, and histopathology reported tumor-free margins with no evidence of local invasion.

## Conclusions

Primary BCC of the conjunctiva is rarely encountered and can resemble OSSN. Therefore, BCC should be considered in the differential diagnosis of patients presenting with atypical symptoms of OSSN. Timely histopathological examination of suspicious lesions is crucial. Complete excision with tumor-free surgical margins may be sufficient, but may also be combined with adjunctive therapeutic modalities, which may enhance treatment in cases of incomplete surgical removal.
